# Proliferative and Apoptotic Pathways in the Testis of Quail *Coturnix coturnix* during the Seasonal Reproductive Cycle

**DOI:** 10.3390/ani11061729

**Published:** 2021-06-09

**Authors:** Sara Falvo, Luigi Rosati, Maria Maddalena Di Fiore, Federica Di Giacomo Russo, Gabriella Chieffi Baccari, Alessandra Santillo

**Affiliations:** 1 Dipartimento di Scienze e Tecnologie Ambientali, Biologiche e Farmaceutiche, Università degli Studi della Campania “Luigi Vanvitelli”, 81100 Caserta, Italy; sara.falvo@unicampania.it (S.F.); mariamaddalena.difiore@unicampania.it (M.M.D.F.); federica.digiacomorusso@unicampania.it (F.D.G.R.); gabriella.chieffi@unicampania.it (G.C.B.); 2 Dipartimento di Biologia, Università degli Studi di Napoli “Federico II”, 80138 Napoli, Italy; luigi.rosati@unina.it

**Keywords:** quail, testis, estrogens, estrogens receptor α, ERK, Akt, PCNA, Bax, cytochrome c

## Abstract

**Simple Summary:**

The quail *Coturnix coturnix* exhibits an annual cycle of testis size, sexual steroid production, and spermatogenesis. The testicular levels of both 17β-estradiol (E_2_) and androgens are higher during the reproductive period compared to the non-reproductive period, suggesting that estrogens act in synergy with the androgens for the initiation of spermatogenesis. Therefore, the present study aimed to investigate the estrogen responsive system in quail testis in relation to the reproduction seasons, with a focus on the molecular pathways activated in both active and regressive quail testes. The results indicated that estrogens participated in the activation of mitotic and meiotic events during the reproductive period by activating the ERK1/2 and Akt-1 pathways. In the non-reproductive period, when the E_2_/ERα levels are low, ERK1/2 and Akt-1 pathways remain inactive and apoptotic events occur. Our results suggest that the activation or inhibition of these molecular pathways plays a crucial role in the physiological switch “on/off” of the testicular activity in male quail during the seasonal reproductive cycle.

**Abstract:**

The quail *Coturnix coturnix* is a seasonal breeding species, with the annual reproductive cycle of its testes comprising an activation phase and a regression phase. Our previous results have proven that the testicular levels of both 17β-estradiol (E_2_) and androgens are higher during the reproductive period compared to the non-reproductive period, which led us to hypothesize that estrogens and androgens may act synergistically to initiate spermatogenesis. The present study was, therefore, aimed to investigate the estrogen responsive system in quail testis in relation to the reproduction seasonality, with a focus on the molecular pathways elicited in both active and regressive quail testes. Western blotting and immunohistochemistry analysis revealed that the expression of ERα, which is the predominant form of estrogen receptors in quail testis, was correlated with E_2_ concentration, suggesting that increased levels of E_2_-induced ERα could play a key role in the resumption of spermatogenesis during the reproductive period, when both PCNA and SYCP3, the mitotic and meiotic markers, respectively, were also increased. In the reproductive period we also found the activation of the ERK1/2 and Akt-1 kinase pathways and an increase in second messengers cAMP and cGMP levels. In the non-reproductive phase, when the E2/ERα levels were low, the inactivation of ERK1/2 and Akt-1 pathways favored apoptotic events due to an increase in the levels of Bax and cytochrome C, with a consequent regression of the gonad.

## 1. Introduction

In seasonal breeding vertebrates, steroidogenesis and spermatogenesis undergo fluctuations during the transition between the stages of the annual cycle [1,2,3]. Sex steroid hormones, such as testosterone and estrogens, are the key factors in the hormonal interplay that governs seasonal breeding [1,2,3]. Estrogens, which have historically been considered female hormones, have exhibited potential functions in the male reproductive system as well, such as in the processes of spermatogenesis and spermiogenesis [4]. The estrogen 17β-estradiol (E_2_) is synthesized by the irreversible conversion of testosterone in the presence of the P450 aromatase enzyme [5]. The action of E_2_ is mediated by two intracellular estrogen receptors (ERs), namely, ERα and ERβ [6]. The information regarding the ERs in avian testis is scarce. Earlier studies have reported the mRNA expression of testicular ERs in the embryos of chicken [7,8] and duck [9], and the adults of gander [10] and Japanese quail [11,12]. The immunoreactivity of ERs has been detected in the testes of chickens [13,14], Japanese quail [15,16], and domestic goose [17]. Meanwhile, a seasonal pattern of ER expression and E2 concentration has only been demonstrated in male domestic goose [17].

Several studies suggested that estrogen activity within the testis might be mediated by the regulation of the activity of mitogen-activated protein kinases (MAPKs) [18,19], which are known to occupy a focal point in signal transduction [20]. The most extensively studied members of the MAPK family are: (1) the extracellular signal-regulated kinases (ERK1 and ERK2), which are involved in the regulation of spermatogonia proliferation and the meiotic progression of spermatocytes [21,22,23], and (2) the serine/threonine kinase Akt-1, which mediates the growth factor-dependent cell survival in various cell types through the inactivation of several pro-apoptotic molecules [24,25]. In the testes of mammalian as well as non-mammalian seasonal breeders, cell proliferation and apoptosis occur at different time points during the annual cycle [26,27,28]. In several bird species, the transition from the breeding to non-breeding stage leads to significant testicular atrophy, which has been associated with the increased incidence of cell death through apoptosis and the decreased rate of cell proliferation [29,30,31,32]. However, the intracellular signal transduction mechanisms underlying these changes occurring in the avian testicular activity during the reproductive cycle remain unknown so far. In this regard, the quail *Coturnix coturnix* represents an excellent avian model, as it demonstrates an annual cycle of testis size, sexual steroid production, and spermatogenesis [33,34,35]. A previous study by our research group revealed that in quail testis, the levels of both E_2_ and androgens were higher during the reproductive period compared to the non-reproductive period, suggesting that estrogens act in synergy with androgens for the initiation of spermatogenesis [35]. Therefore, the present study was aimed to investigate the estrogen responsive system in quail testis in relation to reproduction seasonality. In order to achieve this objective, an analysis of the ERα signaling and the potential participation of ERK1/2 and Akt-1, in both active and regressive quail testes was performed, with particular focus on the proliferative and apoptotic pathways.

## 2. Materials and Methods

### 2.1. Animals

Adult males of quails, *C. coturnix*, (3-month-old) were collected in the surroundings of Naples during January–February (non-reproductive period) and July–August (reproductive period), according to the Ministry of Health guidelines as previously reported [36]. For each period, 10 animals were used. For each animal, the testes were dissected out, weighed and rapidly immersed either in Bouin’s fluid (Sigma-Aldrich, Milan, Italy) and then embedded in paraffin, for histological investigations, or in liquid nitrogen and then stored at −80 °C for biochemical analysis.

### 2.2. 17β-Estradiol Assay

17β-estradiol levels were determined in the testis of quails from both non-reproductive and reproductive periods using an enzyme immunoassay kit (DiaMetra, Milan, Italy). Testes (*n* = 5 from each period) were homogenized 1:10 (*w/v*) with PBS ×1. The homogenate, obtained from each testis, was then mixed vigorously with ethyl ether (1:10 *v/v*) and the ether phase was withdrawn after centrifugation at 3000× *g* for 10 min. The upper phase (ethyl ether) was transferred to a glass tube and was left to evaporate on a hot plate at 40–50 °C under a hood. The residue was dissolved in 0.25 mL of 0.05 M sodium phosphate buffer, pH 7.5, containing bovine serum albumin at a concentration of 10 mg/mL, and then utilized for the ELISA assay [37,38,39]. All samples (three replicates for each sample) ran in the same ELISA. The used 17β-estradiol ELISA kit has the following characteristics: sensitivity of 8.68 pg/mL, an intra-assay variability < 9% and an inter-assay variability < 10%.

### 2.3. Immunohistochemistry

Quail testes from reproductive (*n* = 5) and non-reproductive (*n* = 5) periods were embedded in paraffin. Each paraffin-embedded reproductive and non-reproductive quail testis was cut (5 μm-thick), and the serial sections mounted on poly-L-lysine slides were processed for immunohistochemistry [40,41]. Briefly, deparaffinized sections were washed in 0.1 M phosphate-buffered saline (PBS; pH 7.6), and then incubated in 2.5% H_2_O_2_. To reduce no-specific background the sections were incubated in normal goat serum (Pierce, Rockford, IL, USA) for 1 h at room temperature. Next, sections were treated overnight at 4 °C with the primary antibodies diluted in normal goat serum: (i) rabbit polyclonal antibody ERα (1:400, Abcam, Cambridge, MA, USA), and (ii) mouse monoclonal antibody PCNA (1:300, Sigma-Aldrich, Milan, Italy). After washing in PBS, the sections were incubated for 1 h at room temperature with a biotin-conjugated goat anti-rabbit/mouse secondary antibody (1:2000; Santa Cruz Biotechnology, Inc., Santa Cruz, CA, USA) and an avidin-biotin-peroxidase complex (ABC immune peroxidase kit, Pierce, Rockford, IL, USA), using DAB (Sigma Aldrich, Milan, Italy) as chromogen. Sections were counterstained with Mayer’s hematoxylin. For negative controls, the primary antibody was omitted. Immunohistochemical signal was observed using a Zeiss Axioskop microscope; images were acquired by using AxioVision 4.7 software (Zeiss, Oberkochen, Germany). The immunostaining for both ERα and PCNA was carried out at the same time. Three investigators individually blindly rank the intensity of staining using a light microscope; afterwards, one researcher took digital photographs.

### 2.4. cAMP and cGMP Enzyme Immunoassay

The second messengers, cAMP and cGMP, play a key role in the signal transduction implicated in the regulation of spermatogenesis [42,43]. Frozen testes from reproductive (*n* = 5) and non-reproductive (*n* = 5) quails were weighed and homogenized in 10 volumes of cold 5% TCA. Then, the homogenate obtained from each testis was centrifuged at 600× *g* for 10 min; the supernatant was extracted with 3 volumes of water-saturated ether and aqueous extract was dried. Samples were reconstituted with 0.6 mL of assay buffer 2, a buffer containing proteins, detergents and sodium azide as preservative (A5219, Sigma Aldrich, St. Louis, MO, USA) and then utilized for cAMP (CA201) and cGMP (CG201) enzyme immunoassay (Sigma Aldrich, St. Louis, MO, USA). The acetylated version of both kits was running and the sensitivities were 0.039 pmol/mL for cAMP and 0.088 pmol/mL for cGMP.

### 2.5. Protein Extraction and Western Blot Analysis

Western blot analysis has been performed to investigate the ERα protein expression as well as ERK1/2 and Akt-1 phosphorylations, in both active and regressive quail testes. To determine the testicular proliferative rate in the quail during both reproductive and non-reproductive periods we investigated the protein expression of proliferating cell nuclear antigen (PCNA), a marker of DNA synthesis [44]. SYCP3, encoding the synaptonemal complex proteins and expressed at the meiotic prophase, has been used as meiotic marker [45,46]. Finally, to study the apoptosis pathway, protein levels of Bax and cytochrome c were assayed [47].

Each testis from reproductive (*n* = 5) and non-reproductive (*n* = 5) quails were homogenized directly in lysis buffer containing 50 mM HEPES, 150 mM NaCl, 1 mM EDTA, 1 mM EGTA, 10% glycerol, 1% Triton X-100 (1:2 *w/v*), 1 mM phenylmethylsulphonyl fluoride (PMSF), 1 μg aprotinin, 0.5 mM sodium orthovanadate, and 20 mM sodium pyrophosphate, pH 7.4 (Sigma Chemical Corporation, St. Louis, MO, USA), then clarified by centrifugation at 14,000× *g* for 10 min [35]. Protein concentration was determined by the Bradford assay (Bio-Rad, Melville, NY, USA). Fifty micrograms of total protein extracts for each sample (reproductive and non-reproductive testes) were boiled in Laemmli buffer for 5 min at 95 °C before each electrophoresis. Three electrophoresis sodium dodecyl sulfate–PAGE (12% polyacrylamide) were performed at same time. After electrophoresis, proteins were transferred onto a nitrocellulose membrane. The complete transfer was assessed using prestained protein standards (Bio-Rad, Melville, NY, USA). Each membrane was first treated for 1 h with blocking solution (5% no-fat powdered milk in 25 mM Tris, pH 7.4; 200 mM NaCl; 0.5% TritonX-100, TBS/Tween) and then incubated overnight at 4 °C with one of the following primary antibodies: anti-ERα, raised in rabbit, diluited 1:1000 (Abcam, Cambridge, MA, USA), anti-P-ERK1/2, raised in rabbit, diluited 1:1000 (Cell Signaling, Danvers, MA, USA), anti-ERK1/2, raised in rabbit, diluited 1:1000 (Santa Cruz Biotechnology, Inc., Santa Cruz, CA, USA), anti-P-Akt-1, raised in rabbit, diluited 1:1000 (Cell Signaling, Danvers, MA, USA), anti-Akt-1, raised in rabbit, diluited 1:1000 (Cell Signaling, Danvers, MA, USA), anti-PCNA, raised in mouse, diluited 1:1000 (Sigma-Aldrich, Milan, Italy), anti-SYCP3, raised in mouse, diluited 1:250 (Santa Cruz Biotechnology, Inc., Santa Cruz, CA, USA) anti-bax, raised in rabbit, diluited 1:1000 (Santa Cruz Biotechnology, Inc., Santa Cruz, CA, USA), anti-cytochrome complex, raised in rabbit, diluited 1:1000 (Cell Signaling, Danvers, MA, USA). After washing with TBS-tween, membranes were incubated with horseradish-peroxidase anti-rabbit IgG or anti-mouse IgG secondary antibodies for 1 h at room temperature, followed by signal detection using enhanced chemiluminescence (ECL) (Amersham Bioscience, Bath, UK). The number of proteins was quantified using Image J software (National Institutes of Health, Bethesda, Rockville, MD, USA) and normalized with respect to β-actin protein (housekeeping protein), whose expression did not change between two examined periods.

### 2.6. Statistical Analysis

The values obtained were compared by Student’s *t*-test for between-group comparisons. The differences were considered statistically significant at *p* < 0.05 (*) and *p* < 0.01 (**). All data were expressed as the mean ± standard deviation (SD).

## 3. Results

### 3.1. E_2_ Levels and ERα Protein Expression

**Figure 1:**
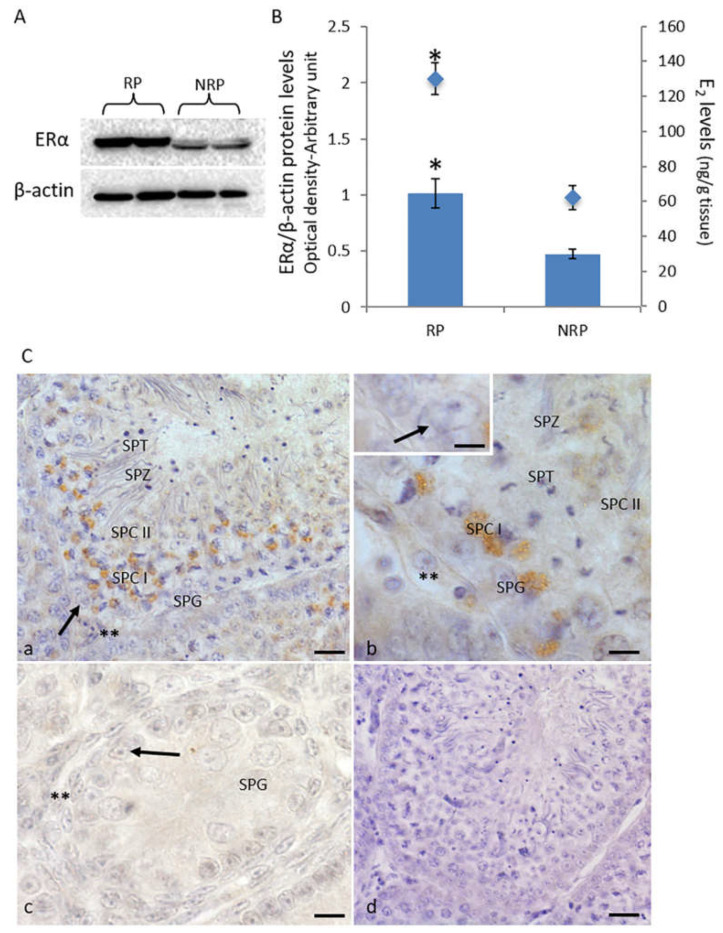
(**A**) Western blot detection of ERα in quail testis during reproductive and non-reproductive periods. A specific band of 66 kDa was detected. (**B**) The amount of protein was quantified using the ImageJ program and normalized with respect to β-actin protein. The values presented represent the means ± S.D. of the values obtained for five animals (two bands were shown). * *p* < 0.05. RP, reproductive period; NRP, non-reproductive period. (**C**) Immunohistochemical localization (brown areas) of ERα. (**a**,**b**) Reproductive period. The signal is evident in Leydig cells (**), spermatocytes I (Spc I) and II (Spc II), spermatids (Spt), and spermatozoa (Spz). No signal is evident in Sertoli cells (arrow) and spermatogonia (Spg). (**c**) Non-reproductive period. The signal is evident in Sertoli cells (arrow). No positivity was observed in Leydig cells (**) and spermatogonia (Spg). (**d**) Control section. Scale bars correspond to 5 µm in (**a**,**c**,**d**) and 10 µm in (**b**).

E_2_ concentration and ERα protein expression in quail testis were analyzed during reproductive as well as non-reproductive periods (Figure 1). In particular, E_2_ levels were observed to be significantly higher in the testes during the reproductive period compared to the values detected in the gonads of non-reproductive quails (130 ± 9 pg/g tissue and 62 ± 7 pg/g tissue, respectively) (Figure 1B).

Western blot analysis revealed that the ERα expression in the testis was significantly higher in the reproductive period compared to the non-reproductive period (Figure 1A,B). These data were supported by the findings of immunohistochemical studies, which revealed an increased signal of ERα in quail testis during the reproductive period (Figure 1C). Specifically, it was observed that during the reproductive period, ERα was localized mainly in Leydig and germ cells, such as spermatocytes I and II, spermatids, and spermatozoa, with the strong positivity detected in spermatocytes I (Figure 1Ca,b). No signal was detected in Sertoli cells and spermatogonia. In the non-reproductive period, a faint positivity was observed only in Sertoli cells (Figure 1Cc). No immunohistochemical signal was detected in the control sections (Figure 1Cd).

### 3.2. cAMP and cGMP Levels

The levels of cAMP and cGMP in quail testes were analyzed in both reproductive and non-reproductive periods. Concentrations of both cAMP and cGMP were observed to be higher in the reproductive period compared to the non-reproductive period (Table 1).

### 3.3. ERK1/2 and Akt-1 Activities

**Figure 2:**
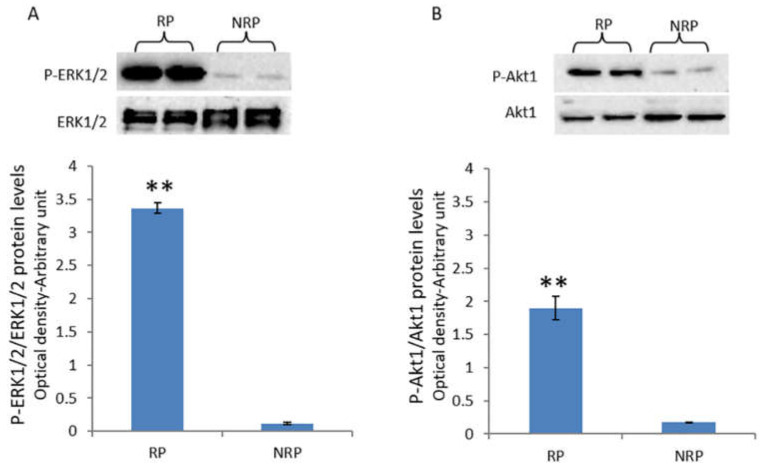
Expressions of P-ERK1/2 and P-Akt-1 in quail testis during reproductive and non-reproductive periods. (**A**) Detection of P-ERK1/2 protein using Western blot analysis. A specific band of 42 kDa was detected. The amount of phosphorylated ERK1/2 was quantified using the ImageJ program and normalized with respect to ERK1/2. The values presented represent the means ± S.D. of the values obtained for five samples (two bands were shown in the upper panel). ** *p* < 0.01. (**B**) Western blot analysis for Akt-1 protein in quail testis during reproductive and non-reproductive periods. A specific band of 60 kDa was detected. The amount of phosphorylated Akt-1 was quantified using the ImageJ program and normalized with respect to Akt-1. The values presented represent the means ± S.D. of the values obtained for five samples (two bands were shown). ** *p* < 0.01. RP, reproductive period; NRP, non-reproductive period.

ERK1/2 activity, expressed as phosphorylated-ERK1/2 (P-ERK1/2) levels, was significantly higher in the reproductive period compared to the non-reproductive period (Figure 2A). Similarly, during the reproductive period, the quail testes exhibited a significant increase in the Akt-1 activity (Figure 2B).

### 3.4. PCNA Protein Expression and Immunolocalization

**Figure 3:**
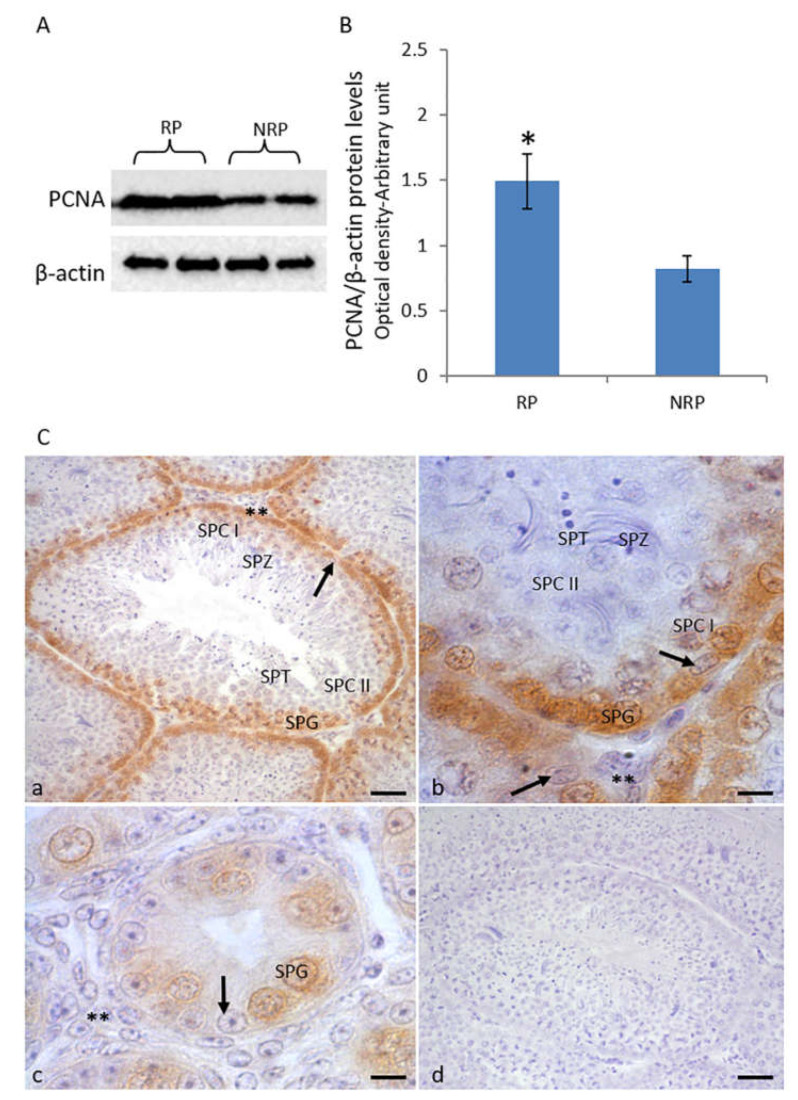
(**A**) Western blot analysis of testicular PCNA expression in quail during reproductive and non-reproductive periods. A specific band of 36 kDa was detected. (**B**) The amount of PCNA was quantified using the ImageJ program and normalized with respect to β-actin. The values presented represent the means ± S.D. of the values obtained for five samples (two bands were shown). * *p* < 0.05. RP, reproductive period; NRP, non-reproductive period. (**C**) Immunohistochemical localization (brown areas) of PCNA. (**a**,**b**) Reproductive period. The signal is evident in spermatogonia (Spg), spermatocytes I (Spc I), and Sertoli (arrow) cells. No signal is evident in Leydig cells (**), spermatocytes II (Spc II), spermatids (Spt), and spermatozoa (Spz). (**c**) Non-reproductive period. The signal is evident only in spermatogonia (Spg). No positivity was detected in Leydig cells (**) and Sertoli cells (arrow). (**d**) Control section. Scale bars correspond to 5 µm in (**a**,**c**,**d**) and 10 µm in (**b**).

Western blot analysis revealed that during the reproductive period, the PCNA protein levels in the testis were higher than those in the non-reproductive period (Figure 3A,B). Immunohistochemical analysis also revealed a more intense immunopositivity for PCNA in the reproductive period with respect to the non-reproductive period (Figure 3). In both periods, a strong immunohistochemical signal for PCNA was detected in spermatogonia (Figure 3Ca–c), while no signal was detected in Leydig cells (Figure 3Ca–c). In the reproductive period, a faint signal was also detected in Sertoli cells and spermatocytes I (Figure 3Ca,b). No immunohistochemical signal was detected for the control sections (Figure 3Cd).

### 3.5. SYCP3 Protein Expression

**Figure 4:**
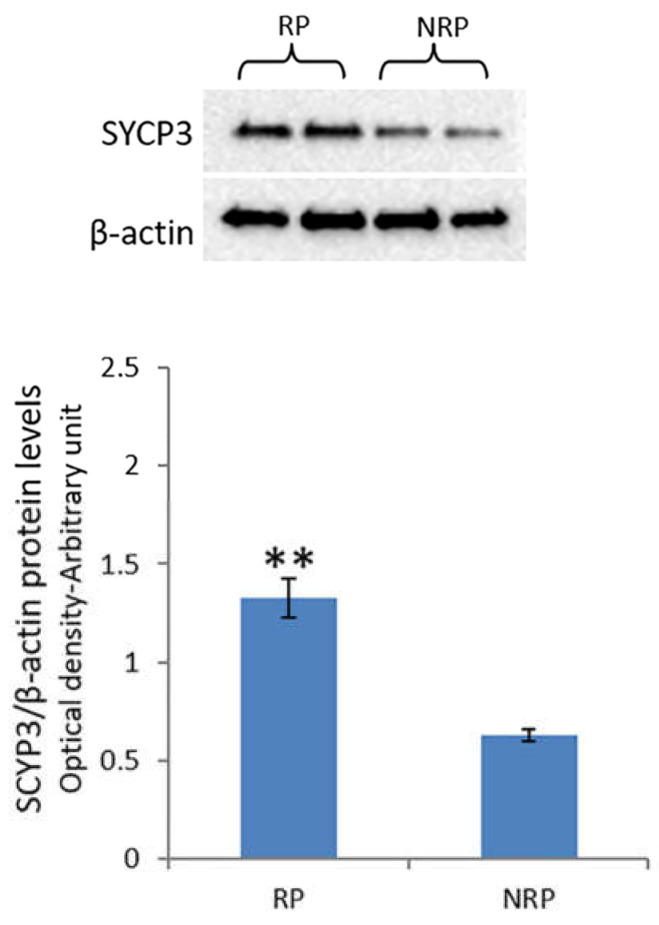
Western blot detections for SYCP3 in quail testis during reproductive and non-reproductive periods. A specific band of 33 kDa was detected. The amount of protein was quantified using the ImageJ program and normalized with respect to β-actin protein. The values presented represent the means ± S.D. of the values obtained for five animals (two bands were shown). ** *p* < 0.01 for reproductive period vs. non-reproductive period. RP, reproductive period; NRP, non-reproductive period.

The expression levels of SYCP3 protein in quail testis during the reproductive period were approximately two-fold higher than those during the non-reproductive period (Figure 4).

### 3.6. Bax and Cytochrome C Protein Expressions

**Figure 5:**
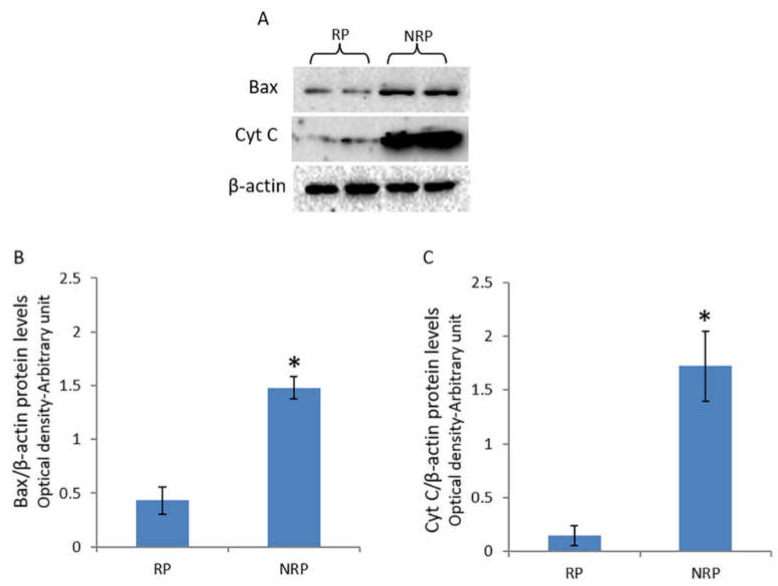
Bax and cytochrome c protein levels in quail testes during reproductive and non-reproductive periods. (**A**) Western blot analysis for Bax (23 kDa) and cytochrome c (14 kDa) proteins in quail testis during reproductive and non-reproductive periods. The amounts of Bax (**B**) and cytochrome c (**C**) were quantified using the ImageJ program and normalized with respect to β-actin. The values presented represent the means ± S.D. of the values obtained for five samples (two bands were shown). * *p* < 0.05 for reproductive period vs. non-reproductive period. RP, reproductive period; NRP, non-reproductive period.

The present study revealed that the levels of both Bax and cytochrome c proteins were significantly higher in the testis during the non-reproductive period with respect to the reproductive period (Figure 5A–C).

## 4. Discussion

It is well established that estrogen signaling is required for the maintenance of male reproductive function in vertebrates [17,21,22,48]. The present study is the first to investigate the E_2_/ERα-activated molecular pathways underlying the testicular activity changes in *C. coturnix* during its seasonal reproductive cycle. Several studies have reported ERα as the predominant form in quail testis [11,15,49], although there is no study on the seasonal pattern of ERα expression in the current literature. In line with our previous study, the present study demonstrated that quail testis exhibits the highest titers of E_2_ during the reproductive period, when P450 aromatase, the enzyme that converts testosterone into E_2_, was expressed at the highest levels [35]. Accordingly, during this period, the levels of both cAMP and cGMP were increased. It is well recognized that both cAMP and cGMP play key roles in spermatogenesis, including the regulation of the blood-testis barrier [43,50]. Furthermore, several studies have demonstrated cAMP to be the main intracellular messenger mediating the expression of P450 aromatase [50], which induces E_2_ production, along with a consequent upregulation of its receptor. The Western blotting and immunohistochemistry analyses conducted in the present study revealed that the expression of the ERα protein was correlated with E_2_ concentration. Specifically, higher levels of ERα protein were detected in quail testis during the reproductive period compared to those in the non-reproductive period. ERα immunopositivity was observed in Leydig cells as well as germ cells (spermatocytes I and II, spermatids, and spermatozoa) during the reproductive period, while a weak immunohistochemical signal was detected only in Sertoli cells during the non-reproductive period. These findings suggest that testicular E_2_ concentration could affect the expression of ERα and that E_2_/ERα levels have a role in the testicular seasonal activity in *C. coturnix*. In particular, the increased levels of E_2_-induced ERα could have a key role in the resumption of spermatogenesis during the reproductive period, when both mitosis and meiosis, as well as the differentiation process of germ cells, are activated. Therefore, we have hypothesized that estrogens might act in synergy with androgens, with the levels of the latter also highest during the reproductive period. In contrast, during the non-reproductive period, when both E_2_ titers and ERα protein expression were significantly reduced, spermatogenesis was blocked. Seasonal fluctuations have also been described in gander testis [16], although in this species the seasonal expression pattern of ERs is different from those of quail. In fact, in gander testis E_2_ levels and its receptors showed an opposite trend, with the highest E_2_ levels in the breeding stage and the highest gene and protein expressions of ERs during the non-breeding stage [16]. The authors hypothesized that excessive doses of estrogens could disrupt testis physiology, therefore a downregulation of ERs protein by E_2_ as a physiological mechanism decreasing the sensitivity of testicular tissue to estrogens has been proposed for gander testes during the breeding season, when the intratesticular E_2_ concentration was very high [16]. A positive correlation between ERα protein expression and spermatogenic activity was reported in the testes of immature, mature, as well as aged chickens [13,51]. Furthermore, in quail, a decrease in both oxidative stress-induced plasma estradiol and ERα testicular expression was reported to reduce spermatogenesis [15]. Different from birds, in the amphibian *Pelophylax esculentus* and reptile *Podarcis sicula*, the estrogenic pathway is essential during the post-reproductive period. It has been proposed that in these species, estradiol mediates the interruption of the reproductive processes inhibiting androgen biosynthesis [39,52,53,54].

Several studies have suggested that estrogen action in the testis might be mediated by the regulation of the activity of mitogen-activated protein kinases (MAPKs) [18,19]. In the present study, the activation of ERK1/2 and Akt-1 pathways in quail testis was investigated. Both ERK1/2 and Akt-1 pathways are reported to play crucial roles in the testis, including the processes of spermatogonia proliferation and meiotic progression of spermatocytes [23,55,56,57,58,59,60]. In the present study, high levels of P-ERK1/2 were detected in quail testis during the reproductive period, while the non-reproductive period testis exhibited considerably low levels of phosphorylated ERK1/2 protein. Concomitantly, the Akt-1 phosphorylation levels were positively correlated with the P-ERK1/2 levels, resulting high in the reproductive period and low during the non-reproductive phase. These findings suggested that during the reproductive period, both ERK1/2 and Akt-1 pathways could be induced by E_2_ via binding to ERα; conversely, in the non-reproductive period, when both E_2_ levels and ERα expression are low, the ERK1/2 and Akt-1 pathways remain inactive. These data are consistent with the other studies, which have reported that E_2_ regulates ERK1/2 and Akt-1 activation in the male germ cells of the frog *Rana esculenta* and lizard *P. sicula* via ERs during the respective annual cycles [21,22,61,62]. A positive correlation between E_2_/ERs signaling and ERK/Akt-1 pathways has also been reported in mammals [63,64]. In vitro experiments have demonstrated a direct role of E_2_/ERs signaling in the stimulation of proliferation in both somatic [18,65] and germ cells [19,66,67,68,69] through the activation of ERK1/2 and Akt-1. Interestingly, it was observed in the present study that the changes in the activation status of both ERK1/2 and Akt-1 were well-correlated with the quail spermatogenetic activity, indicating a key role of these proteins in the regulation of testicular epithelium proliferation and meiotic progression of germ cells in quail testis. In this regard, an increase in the testicular protein levels of PCNA, a mitotic marker, was observed during the reproductive period compared to the non-reproductive period. These findings were supported by the immunohistochemical analysis, which revealed an increased immunohistochemical signal for PCNA in quail testis during the reproductive period. In particular, PCNA was observed to be localized in both somatic and germ cells, with a more intense immunopositivity in spermatogonia. Interestingly, several studies have evidenced that PCNA is regulated by ERK1/2 and Akt-1 signaling pathways. Specifically, the inhibition of ERK1/2 and Akt-1 pathways downregulates the expression of PCNA in the Sertoli cells of chicken [69] and piglets [70], thereby suppressing their proliferation. Recently, it has been demonstrated that treatment with excitatory D-amino acids, which are known to promote spermatogenesis, increases ERK1/2 and Akt-1 activation, as well as the PCNA protein levels, in both frog [71] and rat testis [57] as well as in the spermatogonial cell line GC-1 [58,59,60,72,73]. Chieffi et al. [21,22] reported that the E_2_-induced ERK1/2 activation was associated with the increased PCNA protein expression in the spermatogonia of *P. esculentus* and *P. sicula* during their respective reproductive periods. Therefore, the findings of the present study suggest that in quail testis, PCNA protein expression could be enhanced through the E_2_-induced ERK1/2 and Akt-1 activation during the reproductive period, while in the non-reproductive period, when the ERK1/2 and Akt-1 pathways are inactive, the PCNA protein levels are decreased.

A positive correlation between ERK1/2 and Akt-1 pathway activities and the levels of SYCP3 protein, a meiotic marker, observed in quail testis in the present study indicated a key role of ERK1/2 and Akt-1 phosphorylation in the meiotic progression of germ cells during the reproductive cycle. Similarly, a positive correlation between ERK1/2 activation and the increased number of cells positive for SYCP3 was observed in mouse testis in a previous study [45], which also demonstrated that Akt-1 inhibition abolished SYCP3 induction and the meiotic entry of postnatal mouse male germ cells [56]. Furthermore, inhibition of ERK1/2 was reported to suppress the genic expression of SYCP3 in cultured fetal germ cells [74]. Therefore, the findings of the present study are evidence for the local regulation of quail spermatogenesis via ERK/Akt-1 activation or inhibition during the reproductive cycle.

Studies have reported that reduced PCNA levels in quail testis are associated with testicular regression or atrophy via apoptotic induction [29,44,75,76,77]. Consistent with this, the present study revealed an increase in the expression levels of pro-apoptotic proteins, Bax and cytochrome c, in quail testis during the non-reproductive period; in addition, decreased levels of PCNA and SYCP3 were observed, suggesting that apoptosis might be a factor in the regression of quail testicular activity during the non-reproductive phase of the annual cycle. Interestingly, several studies have reported that apoptosis could be induced by the inhibition of ERK1/2 and Akt-1 signaling pathways [78,79,80]. Therefore, we hypothesized that the inactivation of ERK1/2 and Akt-1 signaling pathways in quail testis during the non-reproductive period could have a key role in the induction of apoptotic events and the consequent regression of the gonad.

## 5. Conclusions

In conclusion, our results indicate that estrogens might be acting in synergy with the androgens in quail testis during the reproductive period. In particular, high levels of E_2_/ERα induce mitotic and meiotic events in germ cells through the activation of ERK1/2 and Akt-1 pathways. On the contrary, during the non-reproductive period, when the E_2_/ERα levels are low, ERK1/2 and Akt-1 pathways are inactive and apoptotic events occur. Therefore, it is suggested that the activation or inhibition of these molecular pathways might have crucial roles in the physiological switch “on/off” of the testicular activity in male quails during their seasonal reproductive cycle.

## Figures and Tables

**Table 1:** cAMP and cGMP levels in reproductive and non-reproductive testes of quail *C. coturnix*.

	Reproductive	Non-Reproductive
cAMP (pmol/g tissue)	45.4 ± 3.6 *	33.6 ± 2.7
cGMP (pmol/g tissue)	31.7 ± 1.2 **	14.5 ± 1.9

** *p* < 0.01 and * *p* < 0.05, reproductive vs. non-reproductive period.

## Data Availability

The data presented in this study are available on request from the corresponding author.
